# Fasting and nutrient-stimulated plasma peptide-YY levels are elevated in critical illness and associated with feed intolerance: an observational, controlled study

**DOI:** 10.1186/cc5127

**Published:** 2006-12-15

**Authors:** Nam Q Nguyen, Robert JL Fraser, Marianne Chapman, Laura K Bryant, Judith Wishart, Richard H Holloway, Michael Horowitz

**Affiliations:** 1 Department of Gastroenterology, Hepatology and General Medicine, Royal Adelaide Hospital, North Terracce, Adelaide, 5000, South Australia, Australia; 2 University Department of Medicine, Royal Adelaide Hospital, North Terracce, Adelaide, 5000, South Australia, Australia; 3 Investigation and Procedures Unit, Repatriation General Hospital, Daws Road, Daw Park, 5041, South Australia, Australia; 4 Department of Anaesthesia and Intensive Care, Royal Adelaide Hospital, North Terracce, Adelaide, 5000, South Australia, Australia

## Abstract

**Introduction:**

Delayed gastric emptying and feed intolerance occur frequently in the critically ill. In these patients, gastric motor responses to nutrients are disturbed. Peptide YY (PYY) slows gastric emptying. The aim of this study was to determine fasting and nutrient-stimulated plasma PYY concentrations and their relationship to cholecystokinin (CCK) in critically ill patients.

**Methods:**

Studies were performed in 19 unselected mechanically ventilated critically ill patients (12 males; 48 ± 7 years old) in a randomised, single-blind fashion. Subjects received a 60-minute duodenal infusion of Ensure^® ^at either 1 or 2 kcal/minute. Blood samples were collected at baseline and at 20, 40, 60, and 180 minutes following commencement of the nutrient infusion for the measurement of plasma PYY and CCK concentrations (using radioimmunoassay). Patient data were compared to 24 healthy subjects (17 males; 43 ± 2 years old).

**Results:**

Fasting PYY concentration was higher in patients (*P *< 0.05), particularly in those with feed intolerance (*P *< 0.05). Plasma PYY concentrations were higher in patients during nutrient infusion (area under the curve [AUC] at 1 kcal/minute: 2,265 ± 718 versus 1,125 ± 138 pmol/l.min, *P *< 0.05; at 2 kcal/minute: 2,276 ± 303 versus 1,378 ± 210 pmol/l.min, *P *= 0.01) compared to healthy subjects. The magnitude of PYY elevation was greater in patients during the 1 kcal/minute infusion (AUC: 441 ± 153 versus 186 ± 58 pmol/l.min, *P *< 0.05), but not the 2 kcal/minute infusion. Fasting and nutrient-stimulated plasma CCK concentrations were higher in patients (*P *< 0.05). There was a relationship between plasma PYY and CCK concentrations during fasting (*r *= 0.52, *P *< 0.05) and nutrient infusion (*r *= 0.98, *P *< 0.0001).

**Conclusion:**

In critical illness, both fasting and nutrient-stimulated plasma PYY concentrations are elevated, particularly in patients with feed intolerance, in conjunction with increased CCK concentrations.

## Introduction

A number of hormones released from the small intestine in response to nutrients modulate gastric emptying and energy intake [1-3]. Peptide YY (PYY) is an important humoral mediator of the entero-gastric feedback mechanism, which leads to a slowing of gastric emptying and small intestinal transit [4-6] and possibly to a suppression of energy intake [1,7,8]. PYY release from the distal small intestine is stimulated both directly by luminal nutrients, particularly the digestion products of fat which activate PYY-secreting cells [7], and indirectly by neuro-endocrine mechanisms, including the release of cholecystokinin (CCK) and insulin-like growth factor-1 [8]. The initial release of PYY after food intake [2,9] is likely to be mediated by CCK [9] and its subsequent release is likely to be mediated through stimulation of PYY-secreting cells by luminal nutrients [9]. Exogenous administration of PYY slows gastric emptying [3,10], which is associated with an inhibition of antral motility [10]. This may contribute to a reduction in energy intake and body weight [11,12]. Similarly, exogenous CCK administration slows gastric emptying [2-4,6], increases the sensation of fullness, and reduces the sensation of hunger and food intake [5,6].

Delayed gastric emptying, which is manifested as intolerance to gastric feeding, is common in critical illness [13,14]. Furthermore, up to 40% of patients suffer from malnutrition during their illness [13-15]. Disturbances in proximal and distal gastric motor activity have been demonstrated in critically ill patients, both during fasting and after enteral nutrient stimulation [16-18]. Recent evidence shows that the entero-gastric feedback response to small intestinal nutrients is elevated in these patients [17], and this elevation may contribute to the disturbances in gastric motility and emptying.

Although the mechanisms underlying enhanced entero-gastric feedback in critical illness are poorly defined, abnormal plasma levels of gut hormones known to modulate nutrient feedback, such as CCK, PYY, and ghrelin, have been demonstrated [19,20]. Our group recently reported that both fasting and nutrient-stimulated plasma CCK concentrations are elevated in critical illness [19], particularly in patients with feed intolerance, suggesting a contribution of this hormone to delayed gastric emptying [19]. Similarly, fasting plasma PYY concentrations are elevated in the first week of admission to the intensive care unit (ICU) and normalise after three weeks [20]. However, not all gut hormone levels are elevated in critical illness. Plasma ghrelin is reduced during fasting, increasing to a normal level as the patient recovers from the illness [20]. There are currently no data regarding the PYY response to small intestinal nutrients or its relationship to CCK in critically ill patients.

The aim of the current study was to assess (a) plasma PYY concentrations during small intestinal nutrient infusion and (b) the relationship between plasma PYY and CCK concentrations in critically ill patients. We hypothesised that plasma PYY concentrations would be elevated in response to duodenal nutrient stimulation, particularly in patients with feed intolerance. Furthermore, because the release of PYY is influenced by CCK, an elevated response would be associated with enhanced CCK secretion.

## Materials and methods

### Study subjects

#### Patients

Studies were performed in 19 critically ill patients (12 males; 48 ± 7 years old; body mass index [BMI] 29.4 ± 2.7 kg/m^2^) who were admitted to a level 3, mixed ICU. Data on fasting and nutrient-stimulated plasma CCK levels in these patients have been published as part of a larger cohort (*n *= 31), including a mixture of critically ill patients with and without prior nutritional support [19]. The patients included in the current study were those who had received prior enteral feeding via a naso-gastric (NG) tube, with sufficient serum to perform the PYY assay. The first criterion was chosen so that the ability to tolerate NG feeds could be determined. All patients had received enteral nutrition (Nutrison Standard: gluten- and lactose-free feed; 100 kcal, 4 g of protein, 12.3 g of carbohydrate, and 3.9 g of fat per 100 ml; Nutricia Nederland N.V., Zoetermeer, The Netherlands) as part of standard clinical care for a mean duration of 3.4 ± 0.9 days. Intolerance of gastric feeding was defined clinically by a gastric aspirate volume of more than 250 ml that was performed every six hours during continuous NG feeding at a rate of more than or equal to 40 ml/hour [21,22]. During both fasting and enteral feeding, all patients received insulin therapy according to a standardised protocol designed to maintain blood glucose concentrations between 6 and 8 mmol/l [16,21].

All patients were at least 18 years old, were mechanically ventilated, and were sedated with propofol (Mayne Pharma Pty Ltd, Melbourne, Victoria, Australia) (10 mg/ml; 1 ml contains approximately 0.1 g of lipid) 24 hours prior to commencement of the study. Exclusion criteria included any contra-indication to passage of an enteral tube; previous gastric, oesophageal, or intestinal surgery; recent major abdominal surgery; and administration of opioid analgesia, benzodiazepine sedative, or prokinetic therapy within 24 hours prior to the study.

#### Healthy subjects

Data were compared with those from 24 healthy volunteers of similar age (17 males; 43 ± 2 years old; BMI 25.5 ± 1.0 kg/m^2^). No subject had evidence of systemic or gastrointestinal disease or was taking any medication known to affect gastrointestinal motility. Healthy subjects were instructed to refrain from smoking during the 24 hours prior to the study.

The study protocol was approved by the Human Research Ethics Committee of the Royal Adelaide Hospital and was conducted according to the National Health and Medical Research Committee Guidelines for the conduct of research on unconscious patients. In patients, written informed consent was obtained from the next of kin prior to enrolment in the study. All healthy subjects provided written, informed consent before entering the study.

### Study protocol

Patients were studied after a minimum of eight hours of fasting. A 12-French 114-cm naso-duodenal feeding tube (Flexiflo; Abbott Ireland Ltd., Dublin, Ireland) was inserted into the distal duodenum via an endoscopically placed guide-wire (THSF-35–260; William A. Cook Australia Pty. Ltd., Brisbane, Australia). Correct placement of the feeding tube in the duodenum was confirmed by (a) measurement of the antro-duodenal trans-mucosal potential difference (TMPD) of greater than -15 mV [17,23] and (b) routine x-ray. Radiologically, this was identified when the tube crossed the midline to the right at the level of the T12 vertebrae and followed the C-shaped curve of the duodenum.

In healthy subjects, the study was performed after an overnight fast of at least eight hours. A silicone rubber catheter (Dentsleeve Ply Ltd., Adelaide, Australia) with a central feeding lumen was used to deliver nutrients into the duodenum. The catheter was inserted transnasally into the stomach and allowed to migrate into the duodenum by peristalsis, without the assistance of either sedation or endoscopy. Passage of the assembly beyond the pylorus was facilitated by small weights located at the catheter tip. Correct positioning of the assembly was determined by continuous measurement of the antro-duodenal TMPD gradient [23]. Radiological confirmation was not performed.

All studies were performed in the morning. Each subject received a 60-minute duodenal infusion of Ensure^® ^(Abbott Laboratories, Abbott Park, IL, USA) (composition: 13% protein, 64% carbohydrate, 21% fat; energy content: 1 kcal/ml) at either 1 or 2 kcal/minute in a randomised, single-blind fashion. Ensure^® ^was diluted with normal saline (0.9%) to 1:4 for the 1 kcal/minute infusion and to 1:2 for the 2 kcal/minute infusion; the resulting solutions were infused at a rate of 240 ml/hour. Blood samples for the measurement of plasma PYY and CCK concentrations were collected at baseline (immediately before nutrient infusion) and at 20, 40, 60, and 180 minutes after commencement of the nutrient infusion.

### Measurement of plasma PYY and CCK concentrations

Blood samples (8 ml) were collected into ice-chilled EDTA (ethylenediaminetetraacetic acid)-treated tubes containing 400 kIU of aprotinin (Trasylol; Bayer Australia Ltd, Pymble, Australia) per millilitre of blood and were centrifuged at 4°C within 30 minutes of collection. The resulting plasma was stored at -70°C for subsequent analysis. Plasma PYY concentrations were measured by radioimmunoassay using an antiserum raised in rabbits against human PYY (1–36) (Sigma-Aldrich, St. Louis, MO, USA) [24]. This antiserum showed less than 0.001% cross-reactivity with human pancreatic polypeptide and sulfated CCK-8 and 0.0025% cross-reactivity with human neuropeptide Y. Tracer (Prosearch International Australia, Malvern, Australia) was prepared by radiolabeling synthetic human PYY (1–36) (Auspep Pty Ltd, Parkville, Australia) using the lactoperoxidase method. Monoiodo-tyrosine-PYY was separated from free iodine-125, diiodo-PYY, and unlabeled PYY by reverse-phase high-performance liquid chromatography (Phenomenex Jupiter C4 300A 5u column cat. no. 00B-4167-EO 250 _ 4.6 mm; Phenomenex Inc., Torrance, CA, USA). Standards (1.6 to 50 fmol/tube) or samples (200 μl of plasma) were incubated in assay buffer (0.05 M phosphate containing 0.5% bovine serum albumin and 0.02% azide [pH 7.4]) with 100 μl of antiserum at a final dilution of 1:10,000 for 20 to 24 hours at 4°C, 100 μl of iodinated PYY (10,000 cpm) was then added, and the incubation continued for another 20 to 24 hours. Separation of the antibody-bound tracer from free tracer was achieved by the addition of 200 μl of dextran-coated charcoal containing gelatin (0.015 g of gelatin, 0.09 g of dextran, and 0.15 g of charcoal per 30 ml of assay buffer), and the antibody-bound tracer was incubated at 4°C for 20 minutes and then centrifuged at 4°C for 25 minutes. Radioactivity of the bound fraction was determined by counting the supernatants in a gamma counter. The intra- and inter-assay coefficients of variation (CVs) were 12.3% and 16.6%, respectively. The minimum detectable concentration was 4 pmol/l [24].

Plasma CCK concentrations were also measured by radioimmunoassay [25]. The antibody (C258; lot 105H4852; Sigma-Aldrich) used binds to all CCK peptides containing the sulfated tyrosine residue in position 7, shows 26% cross-reactivity with unsulfated CCK-8 and less than 2% cross-reactivity with human gastrin, and does not bind to structurally unrelated peptides. The intra- and inter-assay CVs were 9% and 15%, respectively. The detection limit of the assay was 1 pmol/l in plasma [25].

As plasma CCK clearance is primarily dependent on renal function, creatinine clearance in all patients was assessed using the Cockroft-Gault equation and was considered to be impaired when creatinine clearance was less than 65 ml/hr [26].

### Statistical analysis

Data are presented as mean ± standard error of mean. Differences in demographic characteristics and in fasting and integrated (as assessed by area under the curve [AUC] from 0 to 180 minutes) plasma PYY concentrations between critically ill and healthy subjects were assessed using Student's unpaired *t *test. Repeated measures analysis of variance was used to assess (a) differences in nutrient-stimulated PYY responses between the two groups and (b) differences in the PYY response to different nutrient loads within each group. A relationship between plasma PYY and CCK, body weight, APACHE (acute physiology and chronic health evaluation) II score, and length of ICU stay was assessed using Pearson's correlation. A *P *value less than 0.05 was considered significant.

## Results

The study procedures were tolerated well by all subjects and no complications occurred in either group. Nine patients (48.0 ± 6.8 years old) and 11 healthy subjects (43.0 ± 2.0 years old) were randomly assigned to receive a duodenal nutrient load of 1 kcal/minute; 10 patients (51.3 ± 4.4 years old) and 13 healthy subjects (43.1 ± 2.2 years old) received a nutrient load of 2 kcal/minute. The demographic characteristics and admission diagnoses of patients are presented in Table 1. Renal function was impaired in six patients. Eight patients required inotropic support with infusion of either noradrenaline (*n *= 6) or adrenaline (*n *= 2). Ten patients did not tolerate NG feeding before the study. The duration of feeding was similar between feed-tolerant and feed-intolerant patients (3.2 ± 0.7 versus 3.9 ± 1.0 days, respectively).

Over the 24-hour period prior to the study, each patient received an intravenous infusion of propofol (2,110 ± 430 mg) administered with 21.1 ± 4.3 g of lipid as vehicle. There were no differences in either the amount of propofol or related intravenous lipid infused over the 24-hour period between (a) patients who received 1 kcal/minute and 2 kcal/minute infusion or (b) patients with and without feed intolerance.

### Effects of critical illness on fasting PYY concentration

Fasting plasma PYY level was higher in critically ill patients than in healthy subjects (26.7 ± 4.4 versus 16.3 ± 2.0 pmol/l; *P *< 0.05). In patients, fasting PYY concentrations correlated negatively with body weight (*r *= -0.52, *P *= 0.05) and positively with length of stay in ICU (*r *= 0.47, *P *< 0.05). In contrast, there was no correlation between fasting PYY concentration and body weight in healthy subjects. Fasting PYY level in patients with impaired renal function was similar to that of individuals with normal renal function (25.4 ± 9.4 versus 27.3 ± 5.0 pmol/l, *P *= 0.86). There was no correlation between fasting PYY levels and age, gender, or APACHE II score. There was a trend for a higher fasting plasma PYY concentration in patients who received inotropes compared with those who had not received inotropic support (31.2 ± 5.8 versus 23.5 ± 6.0 pmol/l; *P *= 0.125).

### Effects of critical illness on nutrient-stimulated PYY concentrations

**Figure 1:**
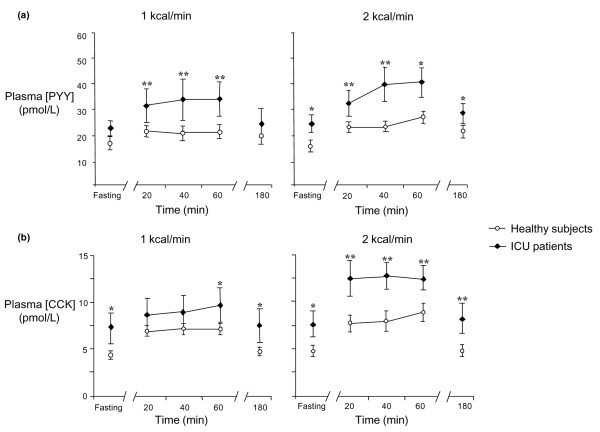
**Effects of critical illness on plasma PYY and CCK concentrations.** Effects of critical illness on plasma **(a) **PYY and **(b) **CCK concentrations during fasting and duodenal nutrient infusions of 1 kcal/minute (ICU patients, *n *= 9; healthy subjects, *n *= 13) and 2 kcal/minute (ICU patients, *n *= 10; healthy subjects, *n *= 11) compared with healthy age- and gender-matched control group. ICU patients: closed diamond, solid line; healthy subjects: open circle, solid line. Fasting and nutrient-stimulated PYY and CCK concentrations were higher in patients compared with the healthy control group. *P < 0.05, **P < 0.001 versus healthy subjects. CCK, cholecystokinin; ICU, intensive care unit; PYY, peptide YY.

In both groups, the absolute plasma PYY concentration increased after 20 minutes of duodenal nutrient infusion and returned to baseline level by 180 minutes. In healthy subjects, the increase in plasma PYY concentration plateaued after 20 minutes (Figure 1a). The magnitude of increase in plasma PYY was greater during the 2 kcal/minute infusion compared with the 1 kcal/minute infusion (AUC _(0–180 min)_: 412 ± 78 versus 186 ± 58 pmol/l.min; *P *< 0.05). In contrast, the increase in plasma PYY concentration in critically ill patients was progressive during the 60-minute nutrient infusion, especially with the 2 kcal/minute infusion (Figure 1a). The magnitude of plasma PYY elevation in patients was comparable between the 1 and 2 kcal/minute infusions (AUC _(0–180 min)_: 441 ± 153 versus 684 ± 258 pmol/l.min).

Both the absolute and integrated plasma PYY concentrations during nutrient stimulation were higher in critically ill patients compared with healthy subjects (Figure 1a; AUC _(0–180 min) _1 kcal/minute: 2,265 ± 718 versus 1,125 ± 138 pmol/l.min, *P *< 0.05; 2 kcal/minute: 2,276 ± 303 versus 1,378 ± 210 pmol/l.min, *P *= 0.01). In patients, there was a greater magnitude of elevation in PYY concentration during the 1 kcal/minute infusion (AUC _(0–180 min)_: 441 ± 153 versus 186 ± 58 pmol/l.min; versus healthy; *P *< 0.05), but not the 2 kcal/minute infusion.

In patients, there was a positive correlation between the magnitude of plasma PYY elevation during nutrient infusion and the fasting concentration (*r *= 0.76, *P *< 0.0001), body weight (*r *= 0.45, *P *< 0.05), and length of ICU stay (*r *= 0.6, *P *< 0.05). There was no relationship between changes in plasma PYY during nutrient stimulation and APACHE II score.

### Effect of critical illness on fasting and nutrient-stimulated CCK concentrations

In both groups, there was an increase in the absolute plasma CCK concentration after 20 minutes of duodenal nutrient infusion. Both fasting and nutrient-stimulated CCK concentrations were higher in patients than in healthy subjects (fasting: 7.3 ± 1.3 versus 4.7 ± 0.5 pmol/l; *P *< 0.05; and nutrient-stimulated: AUC _(0–180 min) _1 kcal/minute: 608 ± 115 versus 395 ± 30 pmol/l.min; *P *< 0.05; 2 kcal/minute: 789 ± 98 versus 518 ± 89 pmol/l.min; *P *= 0.05; Figure 1b). In both groups, the plasma CCK concentration had returned to baseline level by 180 minutes. The magnitude of elevation in CCK concentration was greater in patients during the 2 kcal/minute infusion (AUC _(0–180 min)_: 255 ± 34 versus 176 ± 22 pmol/l.min, *P *= 0.045), but not the 1 kcal/minute infusion (AUC _(0–180 min)_: 110 ± 42 versus 140 ± 26 pmol/l.min, *P *= 0. 42), compared with healthy subjects.

### Feed tolerance and gastro-intestinal hormone response in critical illness

**Figure 2:**
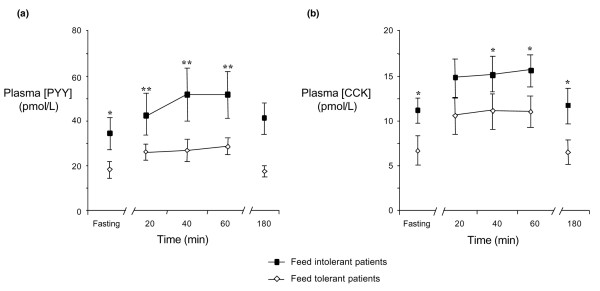
Plasma **(a) **PYY and **(b) **CCK concentrations during fasting and duodenal nutrient stimulation in feed-tolerant (*n *= 9) and feed-intolerant (*n *= 10) critically ill patients. Feed-tolerant patients: open diamond, solid line; feed-intolerant patients: closed square, solid line. Both fasting and nutrient-stimulated PYY and CCK concentrations were higher in patients with feed intolerance compared with feed-tolerant patients. *P < 0.05, **P < 0.001 versus feed-tolerant patients. CCK, cholecystokinin; PYY, peptide YY.

In feed-intolerant patients, both fasting and nutrient-stimulated plasma PYY and CCK concentrations were higher than in the remainder of the group (Figure 2). In feed-intolerant patients, there was a trend for a greater magnitude of elevation in both PYY (AUC _(0–180 min)_: 2,743 ± 589 versus 1,526 ± 335 pmol/l.min; *P *= 0.09) and CCK (AUC _(0–180 min)_: 258 ± 43 versus 148 ± 35 pmol/l.min; *P *= 0.07) concentrations compared with feed-tolerant patients. Both fasting and nutrient-stimulated PYY and CCK levels were similar between feed-tolerant patients and healthy subjects.

### Relationship between plasma PYY and CCK concentrations in critical illness

**Figure 3:**
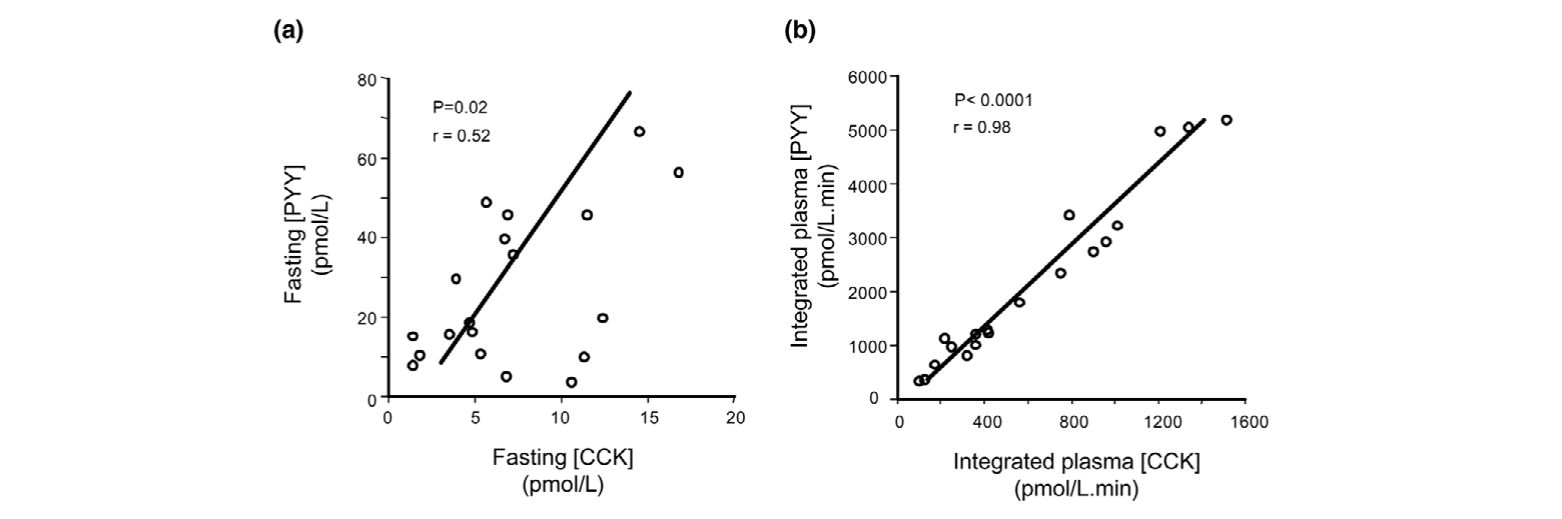
**Relationship between plasma PYY and CCK concentrations during fasting and duodenal nutrient stimulation. **Relationship between plasma PYY and CCK concentrations during **(a) **fasting and **(b) **duodenal nutrient stimulation (expressed as integrated plasma level [area under the curve from 0 to 180 minutes] in critical illness [*n *= 19]). There was a strong positive correlation between integrated PYY and CCK concentrations during both fasting (*r *= 0.52, *P *< 0.05) and nutrient stimulation (*r *= 0.98, *P *< 0.0001) in critically ill patients. CCK, cholecystokinin; PYY, peptide YY.

In patients, there was a strong positive correlation between integrated plasma PYY and CCK concentrations during both fasting (*r *= 0.52, *P *< 0.05) and nutrient stimulation (*r *= 0.98, *P *< 0.0001; Figure 3). In healthy subjects, there was a positive correlation between plasma PYY and CCK concentrations during nutrient stimulation (*r *= 0.43, *P *< 0.05), but not during fasting (*r *= 0.29, *P *= 0.27).

## Discussion

This study is the first to evaluate the plasma PYY response to small intestinal nutrients and its relationship to CCK release in critically ill patients. It was shown that in critical illness, (a) plasma PYY concentrations are elevated during both fasting and nutrient stimulation, particularly in patients who are intolerant to gastric feeding, (b) the release of PYY in response to nutrients does not exhibit the same dose dependency as evidenced in health, and (c) there is a close relationship between nutrient-stimulated plasma PYY and CCK concentrations. These findings are consistent with the concept that the sensitivity of the small intestine to PYY release is increased in critical illness and suggest a potential contribution of this hormone to delayed gastric emptying.

The mechanisms underlying the abnormally high plasma PYY levels during fasting and in response to small intestinal nutrients in critical illness are unclear. In the current study, nutrients were delivered directly into the duodenum to enable a reliable assessment of the entero-gastric feedback response because gastric emptying is frequently disturbed in the critically ill. The use of 1 and 2 kcal/minute nutrient infusions allowed the evaluation of the potential load dependency of the response. The increase in plasma PYY within 20 minutes of nutrient stimulation is most likely mediated by factors in the proximal small intestine rather than by direct nutrient stimulation of the distal ileum. Because prolonged small intestinal transit is common in critically ill patients [27,28], it is unlikely that nutrients would have reached the distal ileum within 60 minutes for direct stimulation of PYY release. This notion is further supported by the subsequent return of plasma PYY to fasting level by 180 minutes, suggesting that the clearance of nutrient from the proximal gut leads to the normalisation of PYY levels.

There are several neuro-hormonal factors related to the proximal small intestine which can potentially elevate PYY concentrations in critical illness. CCK appears to be an important 'proximal' mediator in that (a) it stimulates the release of PYY [8], (b) fasting and nutrient-stimulated plasma CCK are elevated in the critically ill [19], and (c) both fasting and nutrient-stimulated plasma PYY concentrations correlate strongly with CCK. The CCK responses in the current study are consistent with our previous findings [19]. Although the mechanisms underlying the CCK elevation in critical illness remain unclear, recent data suggest that the presence of inflammation, which can influence the entero-endocrine cells in the small intestine, may be important [29]. In a mouse model, McDermott and colleagues [29] demonstrated that upper gut inflammation, via specific control of CD4^+ ^T lymphocytes and related inflammatory cytokines (interleukin-3 and interleukin-4), can upregulate CCK-expressing cells, increase plasma CCK concentrations, and reduce energy intake. Systemic inflammation with elevated inflammatory cytokines is common in critically ill patients [27,28] and may be important in mediating the elevated CCK (and thereby PYY) response to intestinal nutrients. It is also possible that direct neural stimulation in the proximal intestine triggers the release of PYY from the distal ileum [30-32]. In animals, PYY release in response to intestinal nutrients cannot be abolished by preventing nutrient delivery to the site of PYY-containing cells in the distal ileum, but only by removing these cells completely (that is, ileo-colectomy) [31]. These observations suggest the presence of a neural linkage between the proximal gut and the distal PYY-secreting cells, involving sensory vagal fibres with nicotinic, beta-adrenergic, opioid, and serotonergic synapses and nitric oxide release [30-33]. It remains to be evaluated whether disturbances of the neurotransmitters, such as nitric oxide [28], alter the neural function of these pathways and increase small intestinal 'sensitivity' to nutrients in critically ill patients.

Common factors of critical illness such as mechanical ventilation, sedatives, and inotropic therapy may also contribute to the abnormal PYY and CCK responses to nutrients. Apart from inducing splanchnic hypoperfusion, mechanical ventilation with high end-inspiratory pressure can also increase pulmonary and systemic cytokines [28,34,35], which may influence the function of the entero-endocrine cells in the small intestine, as discussed [29]. All patients in the current study were sedated with propofol. Because this agent is administered in a lipid emulsion, a possible direct effect on the hormonal responses cannot be excluded. However, recent data suggest that a lipid amount equivalent to that administered in the current study (20 g) reduced rather than increased PYY concentrations [36]. Similarly, previous reports indicate that intravenous lipid has no effect on plasma CCK concentrations [37-39]. There was a trend for higher plasma PYY concentrations in patients who received inotropic therapy, but whether the elevation is a physiological response to shock or a direct effect of inotropic drugs on PYY release is unknown. In animals, PYY infusion causes intestinal vasoconstriction with a simultaneous increase in systemic arterial blood pressure which serves to divert blood from the gastrointestinal tract to the central vascular system [40]. Further studies are required to assess these associations, in particular whether the heightened PYY response to nutrients predisposes patients to intestinal ischaemia [27].

In the current study, fasting PYY concentrations were 1.5-fold higher in critical illness than in health, consistent with recent findings by Nematy and colleagues [20]. In 16 critically ill patients, these authors found a two-fold increase in fasting plasma PYY concentrations during acute critical illness, with a normalisation of PYY levels after three weeks of admission to the ICU. In these patients, fasting PYY levels were positively related to changes in appetite and energy intake [20], suggesting a relationship between critical illness, elevated plasma PYY concentrations, appetite, and possibly gastric emptying. Our finding of a substantially higher PYY response (approximately two-fold) to small intestinal nutrients in critical illness, particularly in patients with feed intolerance, supports such a relationship and a contribution of PYY to the enhanced entero-gastric feedback response. The lack of a dose-dependent response to nutrients in critically ill patients is consistent with the concept that the 'sensitivity' of the small intestine to PYY release is enhanced. In non-critically ill patients with cardiac cachexia associated with primary pulmonary hypertension, an enhanced 'early' PYY response to an intragastric meal has been reported [41]. Accordingly, our observations provide a potential link between abnormal 'entero-gastric' responses [17] and the frequently observed gastric motor dysfunction and delayed emptying in the critically ill [16-18,21]. Intolerance to gastric feeding is an indirect marker of delayed gastric emptying in these patients [21,22]. Together, these observations support the concept that the humoral mediators of the 'entero-gastric' response are enhanced in critical illness, providing a potential mechanism for delayed gastric emptying and subsequent feed intolerance.

The heterogeneity of the critically ill population limits the interpretation of the study data. In particular, various admission diagnoses that can have an impact on gastric emptying were included in this study. In light of the normalisation of fasting PYY levels in critically ill patients after discharge [21], follow-up studies to examine the long-term recovery of the humoral response and entero-gastric feedback would be important. The decrease in plasma ghrelin during critical illness suggests that the elevated PYY and CCK responses may be a specific phenomenon. The role and specificity of these hormones in feed intolerance can be evaluated further by assessing other gut hormones, such as glucagon-like-peptide 1, secretin, gastric inhibitory polypeptide, neurotensin, and motilin.

## Conclusion

Both fasting and duodenal nutrient-stimulated plasma PYY concentrations are elevated in critical illness, particularly in patients who are intolerant to gastric feeding. This elevated response is strongly related to plasma CCK concentrations, suggesting an important role for this hormone in mediating increased PYY release. Together, these findings provide an underlying humoral mechanism for the enhanced entero-gastric reflex and subsequent delayed gastric emptying in critical illness.

## Key messages

• In critical illness, plasma PYY concentrations are elevated during both fasting and nutrient-stimulation, particularly in feed-intolerant patients.

• In critical illness, the release of PYY in response to nutrients is not dose-dependent.

• In critical illness, there is a close relationship between nutrient-stimulated plasma PYY concentration and CCK concentration.

• These observations support the concept that the humoral mediators of the 'entero-gastric' response are enhanced in critical illness, providing a potential mechanism for delayed gastric emptying and possible targets for therapy.

## Abbreviations

APACHE = acute physiology and chronic health evaluation; AUC = area under the curve; BMI = body mass index; CCK = cholecystokinin; CV = coefficient of variation; ICU = intensive care unit; NG = naso-gastric; PYY = peptide YY; TMPD = trans-mucosal potential difference.

## Competing interests

The authors declare that they have no competing interests.

## Authors' contributions

NN, RF, MC, RH, and MH conceived the study, participated in its design and coordination, and helped to draft the manuscript. NN and LB participated in the study design, carried out the studies and data and statistical analysis, and drafted the manuscript. NN and MC were involved in recruiting patients from the ICU of the Royal Adelaide Hospital. JW performed the radioimmunoassay. All authors read and approved the final manuscript.

## Figures and Tables

**Table 1:** Demographic details and admission diagnoses of critically ill patients

Patient	Age (years)	BMI (kg/m^2^)	Days in ICU	Admission APACHE II	Feed-tolerant	Renal function	Inotrope support	IV propofol (mg/24 hours)	IV lipid associated with propofol (g/24 hours)	Nutrient load	Admission diagnosis
1	36	27	3	26	No	Normal	No	1,200	12.0	1 kcal/minute	Head injury
2	69	31	3	23	Yes	Normal	No	2,400	24.0	1 kcal/minute	Meningitis
3	27	20	10	18	No	Normal	No	6,000	60.0	1 kcal/minute	Head injury
4	66	33	5	26	No	Impaired	No	1,200	12.0	1 kcal/minute	Pancreatitis
5	23	25	5	21	No	Normal	Nor A	720	7.2	1 kcal/minute	Multi-trauma
6	59	19	13	30	Yes	Normal	Nor A	4,800	48.0	1 kcal/minute	Sepsis
7	57	35	7	37	No	Impaired	A	1,200	12.0	1 kcal/minute	Sepsis
8	23	26	10	25	Yes	Normal	No	1,680	16.8	1 kcal/minute	Burn injury
9	72	29	6	21	Yes	Impaired	Nor A	1,200	12.0	1 kcal/minute	Sepsis
10	53	29	3	27	No	Impaired	Nor A	4,800	48.0	2 kcal/minute	Sepsis
11	41	41	9	18	No	Normal	No	1,200	12.0	2 kcal/minute	Sepsis
12	74	35	5	27	Yes	Impaired	A	1,440	14.4	2 kcal/minute	Cardiac failure
13	27	26	2	26	No	Normal	Nor A	480	4.8	2 kcal/minute	Pancreatitis
14	47	27	8	19	Yes	Normal	No	6,000	60.0	2 kcal/minute	Multi-trauma
15	66	34	4	28	No	Impaired	No	1,200	12.0	2 kcal/minute	Cardiac failure
16	55	29	9	30	Yes	Normal	No	480	4.8	2 kcal/minute	SDH
17	36	27	11	29	No	Normal	No	960	9.6	2 kcal/minute	Multi-trauma
18	55	21	7	22	Yes	Normal	No	3,120	31.2	2 kcal/minute	Multi-trauma
19	59	20	6	29	Yes	Normal	Nor A	720	7.2	2 kcal/minute	Sepsis

A, adrenaline; APACHE, acute physiology and chronic health evaluation; BMI, body mass index; ICU, intensive care unit; IV, intravenous; Nor A, noradrenaline; SDH, subdural haemorrhage.
